# Determinants of parent-reported child mental health status in San Diego public schools during the height of the COVID-19 omicron outbreak: A serial cross-sectional study

**DOI:** 10.1371/journal.pone.0288628

**Published:** 2023-07-26

**Authors:** Lotus McDougal, Araz Majnoonian, Gabriela Stone, Rebecca Fielding-Miller

**Affiliations:** 1 Division of Infectious Diseases and Global Public Health, School of Medicine, University of California San Diego, La Jolla, California, United States of America; 2 Herbert Wertheim School of Public Health and Human Longevity Science, University of California San Diego, La Jolla, California, United States of America; Al-Ahliyya Amman University, JORDAN

## Abstract

Despite extensive debate on the effects of COVID-19 mitigation measures in school settings, little evidence exists on trends in school children’s mental health over the course of the COVID-19 pandemic. The objective of this paper was to identify factors affecting parent reports of school children’s mental health during COVID-19 Omicron variant outbreak in a cohort of high-risk, socially vulnerable children attending public elementary schools. We analyzed four waves of cross-sectional, online-administered surveys completed by parents of children attending public elementary schools in San Diego between November 2021 and March of 2022. Children (n = 684) ranged in age from 2–17 years. We used multilevel linear mixed effects models to assess determinants of parent-reported child mental health status. The outcome was child mental health, as reported by the parent. Parents consistently rated their children’s mental health as very good, though parents who experienced recent COVID-related challenges and who had older children reported lower levels of mental health in their children. Children’s mental health was generally considered to be very good, as judged by their parents during a period of constant in-school masking and the Omicron variant outbreak. Structural support mechanisms aimed at mitigating COVID-related challenges for adults may offer benefit to children’s mental health.

## Introduction

The COVID-19 pandemic has profoundly impacted morbidity and mortality, including mental health [1,2]. Depression and anxiety increased during COVID-19, with particularly acute increases early on when information on the COVID-19 virus was limited and social isolation measures were more common [3,4]. Few populations have been unaffected, with substantial adverse mental health effects documented among children and adolescents [4–7]. In the United States, the pandemic has disproportionately affected communities made socially vulnerable by decades of exposure to institutional racism and community disinvestment [8,9]. These intersectional vulnerabilities have manifested in elevated levels of adverse mental health during the COVID-19 pandemic among girls, and children in families who are socioeconomically disadvantaged, or with only single parents [10].

The majority of mental health-focused COVID research published to date has focused on earlier phases of the pandemic [11], with less understanding of how the protracted and oscillating nature of infection levels, virulence, and protection measures have impacted mental health [10,12]. Existing research on the influence of mitigation measures such as quarantine on mental health confirms adverse effects, but is lacking evidence on the mental health impacts on children [13]. Moreover, while the effects of the pandemic and pandemic-related mitigation measures have been the subject of much political debate, relatively little actual evidence exists on trends in child mental health outcomes among children whose communities have been the most directly affected [14,15].

By spring 2022, most children in the United States had experienced a variety of remote, hybrid, and in-person school modalities, which differentially affect mental health. While the experience of remote or hybrid education may be stressful and isolating, the visibility of non-pharmaceutical measures during in-person schooling (i.e., distancing, masking) serves as a visible cue of the ongoing pandemic [16]. Decreased vaccine uptake has also been associated with higher levels of worry in children across the United States [17]. This study aims to measure levels of mental health among school-age children in San Diego during the height of the Omicron outbreak during a period of constant in-school mask mandates, to assess factors influencing mental health in this population of children.

## Methods

### Study design and setting

This serial cross-sectional study comprised 42 school sites enrolled in the Safer at School Early Alert (SASEA) program in the 2021–2022 academic year, selected based on a combination of zip code level factors: (1) high social vulnerability according to the California Healthy Places Index, (2) COVID-19 case rates in the top quintile of the county as of August 2021, and/or (3) relatively low rates of vaccine uptake. Three classrooms, sampled with replacement, were randomly selected per school every 4–6 weeks from November 2021 –May 2022 (6 waves).

### Sampling and recruitment

Within the three randomly selected classrooms in each school, students received flyers containing links to self-administered surveys to take home to their parents. Each wave was open for approximately two weeks, and available online in English or Spanish, or over the phone with a trained research assistant. Students were incentivized to pass the flyer along to their parents by providing a gift card for a class pizza party to the class with the highest response rate for each wave.

### Analytic sample

This analysis focuses on surveys completed during mandatory in-school masking, which ended on April 2, 2022. Of the 707 surveys completed between November 2021 –March 2022, 684 parents responded to questions assessing their children’s mental health. Only three parents chose to complete the survey over the phone.

### Measures

The primary dependent variable, parent-reported child mental health (PRCMH) status, was assessed by asking parents “Would you say your child’s mental health in general is excellent, very good, good, fair, or poor?”, with responses coded from excellent = 5 to poor = 1. We chose to use a single item for assessing PRCMH to reduce participant burden. While less nuanced, single-item measures of mental health have generally good correlation with more in-depth scales [18]. Independent variables were determined a priori and included recent COVID-related challenges, child COVID vaccination status (yes, no/ineligible), parent COVID vaccination status (yes/no), child age (years), parent age (years), parent gender (female, male), English spoken in the home (yes, no), number of people in the household, and interview date relative to statewide mask mandate (did not apply to schools; before, during, after) and weekly COVID-19 cases per 100 population in the school’s zip code. COVID-19 cases were obtained from the SanGIS/SANDAG GIS Data Warehouse. COVID-related challenges was an index ranging from 0–10 measuring challenges (0 = no challenge, 1 = minor challenge, 2 = major challenge) across five areas (getting health care, having a place to stay/live, getting enough food, getting needed medicine, and getting to needed locations). Waves were accounted for by inclusion of interview date as a covariate.

### Analysis

Descriptive analyses were conducted to assess the prevalence of all measures in total, and stratified by levels of the dependent variable (PRCMH). PRCMH was also descriptively analyzed to assess trends over time. Multivariable regression analysis was used to assess the influence of potential determinants of PRCHM. We used multilevel linear mixed effects models with random effects for classrooms nested within schools, modelling PRCMH as the dependent variable, and including all independent variables noted above as covariates.

### Ethics

Ethical approval for SASEA data collection was provided by the University of California, San Diego Institutional Review Board (approval 201664). The parent study was reviewed and approved by district leadership in all participating school districts. All survey participants were 18 years of age or above and provided informed written consent through REDCap before starting the survey. Those who completed the survey with an interviewer provided verbal consent, documented by interviewer signature. All survey participants were entered into a raffle to win one of $100 gift cards per survey wave per regional cluster. Preliminary study results were shared with school communities via infographics, townhall meetings, and a dedicated website.

## Results

**Fig 1:**
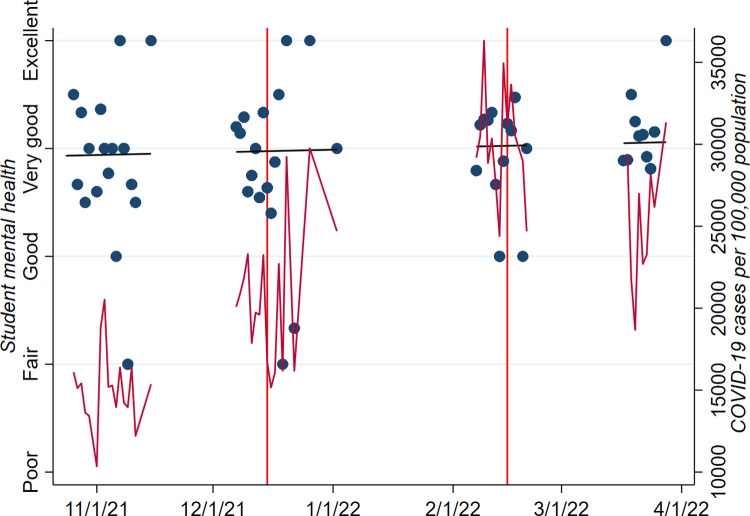
Levels of parent-reported student mental health and COVID-19 prevalence over the survey period. Note: Vertical red lines indicate commencement and cessation of mandatory in-school mask mandate.

PRCMH remained relatively stable between November 2021 and March 2022, despite regular increases in the local prevalence of COVID-19 in the area during this period (Fig 1).

On average, parents rated their children’s mental health as 4.0, equivalent to “very good” (Table 1). Respondents were an average of 36 years of age and 87% female; their children were an average of eight years of age. The majority of respondents (87%) reported receiving COVID-19 vaccinations themselves, but the minority (37%) reported that their children were vaccinated. Most respondents (74%) spoke English at home. When compared to school demographic data provided by the state of California, survey participants were less likely to be Asian or non-Hispanic white (analyses not shown) [19].

**Table 1:** Sample descriptive statistics.

	Total		Child mental health					
	N	%	Mean	Excellent	Very good	Good	Fair	Poor
Total	684	100	4.01	40.2	32.3	17.1	9.1	1.3
Recent COVID-related challenges (mean) ^1^	684	0.1	-	0.1	0.1	0.1	0.2	0.3
COVID vaccination—child								
No	406	63.2	4.1	70.7	60.0	53.7	62.3	44.4
Yes	236	36.8	3.9	29.3	40.0	46.3	37.7	55.6
COVID vaccination–parent								
No	84	12.6	4.1	14.3	11.5	11.4	11.5	11.1
Yes	583	87.4	4.0	85.7	88.5	88.6	88.5	88.9
Child age (mean)	683	8.0	-	7.5	7.9	8.7	8.6	9.7
Parent age (mean)	639	36.3	-	35.6	36.5	37.1	36.2	43.1
Parent gender								
Female	578	87.4	4.0	87.2	88.9	86.6	86.7	75.0
Male	83	12.6	4.0	12.8	11.1	13.4	13.3	25.0
English at home								
No	177	26.1	4.1	28.3	27.4	21.6	22.6	11.1
Yes	501	73.9	4.0	71.7	72.6	78.5	77.4	88.9
People in household (mean)	674	4.9	-	5.0	4.8	4.9	4.7	4.7
Mask mandate								
Before	193	28.2	4.0	30.9	23.5	27.4	37.1	11.1
During	231	33.8	4.0	29.1	39.4	33.3	37.1	22.2
After	260	38.0	4.1	40.0	37.1	39.3	25.8	66.7

^1^COVID-related challenges related to health care, a place to stay/live, food, medicine, and transport in the past 6 months, ranging from 0 (no challenges) to 1 (all major challenges).

Over the survey window, recent COVID-related challenges and child age were significantly and negatively associated with PRCMH (challenges coefficient = -1.05, p<0.001; child age coefficient = -0.05, p = 0.01)(Table 2). Increases in weekly COVID-19 case prevalence were associated with improvements in PRCMH (coefficient = 0.01, p = 0.02). There were no significant associations between other covariates and PRCMH.

**Table 2:** Multilevel linear mixed-effects models assessing factors associated with parent-reported student mental health.

	β	p-value
Time (days)	0.004	0.15
Recent COVID-related challenges^1^	-1.04	<0.001
COVID vaccination—child		
No	REF	
Yes	-0.15	0.15
COVID vaccination–parent		
No	REF	
Yes	0.05	0.70
Child age	-0.05	0.01
Parent age	-0.01	0.28
Parent gender		
Female	REF	
Male	0.17	0.22
English at home		
No	REF	
Yes	-0.15	0.15
People in household	0.01	0.39
Mask mandate		
Before	REF	
During	-0.27	0.14
After	-0.35	0.21
COVID-19 cases per 100 population	0.01	0.03

^1^COVID-related challenges related to health care, a place to stay/live, food, medicine, and transport in the past 6 months, ranging from 0 (no challenges) to 1 (all major challenges).

Model includes random effects for classrooms nested within schools.

## Discussion

Between November 2021 and March 2022, this sample of low-income, socially vulnerable San Diego K-12 parents rated their children’s mental health as “very good”, with no significant change over time. PRCMH thus remained relatively positive overall during this period of mandatory in-school masking and was unaffected by the institution or dissolution of the statewide mask mandate (which was distinct from in-school masking policies).

The largest factor affecting PRCMH in our sample was recent COVID-related challenges, which were most commonly difficulty in earning a stable income and in accessing health care. This is consonant with barriers and hardships faced by other populations during the pandemic [20], and important in underscoring that parents’ mental health can affect their children’s mental health [14,21]. We have found similar results in other vulnerable populations in the region [22–25]. With the lifting of public health emergency measures in the United States, social safety net programs will become less accessible to these same populations [26]. Our findings suggests that this may have major implication for child mental health in the coming years.

Older children had worse PRCMH than younger children in this sample. This finding has emerged in other settings, and may be reflective in part of older children’s increasing developmental and cognitive capacity to understand and be concerned by external stressors [27]. The fact that neither higher levels of parent COVID vaccination nor lower levels of child COVID vaccination, including among children not yet eligible for vaccination, was related to PRCMH, may be related to the fact that by the time this study was fielded vaccine uptake had largely plateaued, and hence become normalized.

Increases in local COVID-19 prevalence were associated with slight improvements in PRCMH during this time. As the study period encompassed the height of the Omicron variant surge of COVID-19 in San Diego, this finding may be reflective of more long-term resilience of mental health status as the COVID-19 pandemic progresses [28], and of school breaks and holiday gatherings which coincided with (and in the latter case, arguably fueled) the Omicron surge.

These results should be interpreted in light of key limitations. Self-report data is subject to social desirability bias. Our outcome of interest, child mental health, is measured based on parent report, and thus subject to proxy bias, though there is evidence to suggest strong correlation between parent and child report [29,30]. Additionally, in-school mask mandates across San Diego County were lifted just after the close of this study window, thus their effects are outside the scope of this analysis.

### Conclusion

Parents in this sample of San Diego public school children rated their children’s mental health as very good overall during a period of constant in-school masking, noting very low levels of adverse mental health during mandatory institutional masking. Rather, children’s mental health was most directly affected by their families having experienced recent COVID-related challenges such as income disruption and health care barriers. This is in line with prior research suggesting that familial structure and stability offer children increased resilience against adverse mental health associated with COVID-19 [10,31]. Offering structural support mechanisms to families facing financial and health care instability may additionally bolster their children’s mental health during this period of tremendous social change.
